# Nested information processing in the living world

**DOI:** 10.1111/nyas.14612

**Published:** 2021-05-26

**Authors:** Tilmann Wurtz

**Affiliations:** ^1^ Independent Scholar Tullinge Sweden

**Keywords:** intracellular signal chains, information processing, cell communication, development, logic gates, interactive networks

## Abstract

Living organisms create, copy, and make use of information, the content depending on the level of organization. In cells, a network of signal chain proteins regulates gene expression and other cell functions. Incoming information is encoded through signal reception, processed by the network, and decoded by the synthesis of new gene products and other biological functions. Signaling proteins represent nodes, and signal transmission proceeds via allosteric binding, chemical and structural modifications, synthesis, sequestering, and degradation. The induction of the gene caudal type homeobox 2 (*CDX2*) in the mammalian preimplantation embryo is outlined as a demonstration of this concept. CDX2 is involved in the decision of cells to enter the trophoblast lineage. Two signal chains are coordinated into an information processing model with the help of logic gates. The model introduces a formal structure that incorporates experimental and morphological data. Above the cell level, information flow relates to tissue formation and functioning, and whole cells play the role of network nodes. This is described for the anatomical patterning of bone with implications for bone formation and homeostasis. The information usage in cells and tissues is set into a context of the nervous system and the interaction of human individuals in societies, both established scenes of information processing.

## Graphical abstract

This article illustrates the informational context of a gene regulation event that is initiated by cell communication. The ramifications of such events into the development of anatomical structures and other biological systems of information processing are outlined. A comparison with the elements of electronic information technology has helped gain insight into biological function, although living cells and tissues follow different and mostly unknown data processing mechanisms.

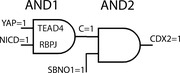

## Introduction

The impact of information processing on cellular regulation has been appreciated, and possible biology‐based systems of information processing have been brought to our attention.^1^ Living beings differ from inanimate nature by their usage of information flow;^2^ and the regulated usage of genetic information is central to their development, maintenance, and propagation.^3^ All biomass is synthesized and assembled by information‐dependent mechanisms.^2^ If biological data are presented around the information flow, certain functional aspects may be visualized in a new way.

For the analysis of the central nervous system, the data processing role is the main theme, and also interhuman interactions have frequently been described in relation to information processing, for example, in *The World Brain* a by H.G. Wells, *The Global Brain* b by P. Russell, or *Collective Intelligence*.^4^ Concerning the lower level of biological organization, the individual cell and the coordination of cells in the development and maintenance of tissues, information flow attracts relatively little attention,^2^, ^3^, ^5^ the main focus of research being on the identification of components and the analysis of their interactions in a linear way. This article presents a gene regulation event to illustrate the informational context of intracellular processes and the development of anatomical structures as an example of cell communication. Reference is made to electronic information technology since data processing of conventional computers is formalized and well understood. Although living systems follow mechanisms of data processing that are mostly unknown, this comparison can help gain insight into biological mechanisms.

## The cell as a computation machine

Communication of computers with the user occurs via screen, printer, keyboard, mouse, microphones, and scanners. Incoming signals are translated into binary code because this allows to perform automatic calculations by electric signals according to Boole's algebra. Data processing is executed in integrated circuits of the motherboard. Integrated circuits contain millions of transistors that act as switches. They are assembled in the form of logic (Boolean) gates, structures that transmit data, filter data propagation, or combine signals. Logic gates and storage elements process data according to stored instructions (programs). A hierarchy of networking can be perceived: transistors are nodes in logic gates, logic gates are nodes in integrated circuits, and integrated circuits are nodes in the motherboard. Data are propagated between nodes, and the propagation of data is influenced by other nodes. The processing sequence is programmable, and the outcome is decoded and displayed or stored. The computing process depends on storage media, often a hard disk or a solid‐state disk for long‐term storage and random‐access memory (RAM) for immediate access. Depending on the program in use, a given input signal will elicit different output results. This description is valid for classical information technology. Self‐learning neural networks may sometimes organize operations in unknown ways.^c^

Living cells also receive signals, encode, and process information, and decode and display responses. Signal reception occurs at the outer cell membrane, while processing is performed inside the cell. Responses are the synthesis and secretion of new macromolecules, which are incorporated or exported, thereby influencing cell properties and, in some cases leading to suicide (e.g., apoptosis). Processing responses change the cell itself; there is no distinction between hardware and software. In fact, any given cell is the product of information processing and synthetic events of its ancestors. The cell's information content is represented by its components and their structural arrangement, for example, the 3D folding of protein chains. Cells also contain genetic information in the form of the sequences of DNA and RNA molecules. Genes encode protein sequences, and proteins serve as building blocks of cellular and extracellular structures, as enzymes that catalyze biological processes, or as regulatory elements. Taken together, the cell embodies information that is at the base of further reception and processing of signals. New signals alter the information content as well as the physical appearance of cells.

Most signals are received at the cell membrane, a lipid layer surrounding the cell body. This layer contains receptor proteins that specifically recognize different cues: cell membrane proteins of neighboring cells, such as integrins, selectins, cadherins, Wnt proteins, extracellular matrix (ECM) components (collagen and others), and diffusible regulatory effectors (growth factors, hormones, vitamins, interferons, interleukins, and cytokines). Receptors, when binding to their targets, trigger chains of reactions inside the cell that propagate signals into the cell nucleus toward the regulation of genes and the management of cell's long‐term memory. Signal chains operate by chemical modification (phosphorylation) and allosteric protein binding, both directing the folding of protein chains in three dimensions. Thereby, the surface of protein molecules is modulated, and their biological activity is altered. Enhancement of signal strength may be achieved if an enzyme is activated, which generates a second messenger like cAMP, or if the influx of Ca^2+^ is triggered by opening membrane channels.^5^ Proteins that constitute the signal chain interact with each other in a sequential way. Signals are passed from one protein molecule to the next, and signal propagation occurs if an element is in an active state. It is possible that activation needs more than one step, but signal transmission is in its essence binary (1 or 0).^2^, ^5^ This article focuses on gene regulation; there are other regulatory pathways of signal propagation that concern metabolism, membrane functions, translation, and so on.^5^

The combined intracellular signal chains represent a functional entity for cells. Their biological role is information processing by organizing the information flow between signal reception and gene regulation—the “organelle” that corresponds to the motherboard of the computer. This argument resides on two lines of evidence outlined below. First, the signal is propagated in a coded form in relation to the extracellular situation since signal propagation inside and outside involves different actors. At reception, incoming signals are coded into an intracellular language. Second, components of many signal chains are interconnected and represent molecular switches. Since the code is binary (active or inactive), components may be assembled into logic gates, where information is processed, and decisions are made.

A network mediation between signal reception and gene regulation is an asset for the cell's information management. If external signals, stimulating or repressing gene expression, would address their target genes directly, a multitude of genes would be influenced by different signals. Many messenger molecules would be needed, and contradictory information would prevail at the gene level. By contrast, the network, or processing unit, summarizes and coordinates all incoming signals. Concerning its biochemical structure, multicomponent complexes (signalosomes) at the signal reception and gene regulation sites were identified,^6^ and signaling complexes in‐between where components are organized by scaffolding proteins.^5^, ^6^ The operative program of this processing unit is constituted by its composition because this decides how signals are propagated. Components are the set of receptors that are displayed, the numerous genetic and splicing variants of all interactive factors,^5^ and the elements of gene regulation sites (see below).

Formation and interaction of signalosomes may involve liquid–liquid phase separations with effects on the interaction kinetics.^6^ The interaction between components follows nonequilibrium dynamics, observed in several model systems.^7^ The composition of signalosomes is cell type–specific and will change during a signaling process. Many copies of each signal chain component exist in each cell, part of them being nonbound and others engaged in different interactions.^6^ On the other hand, logic gates are individual structures with a distinct signal–effect relationship. Therefore, the mapping of protein species to specific logic gates is difficult. The gene regulation site might promise a technical simplification since a diploid cell contains only two copies of most genes, and the direct effect, RNA production, is explicit.

Since the cell membrane contains many different receptors, many signal chains are active at the same time. Further diffusible effectors interact; for example, steroid hormones and second messengers. The same holds for NOTCH signaling, where a receptor fragment, NOTCH1 intracellular domain (NICD), represents the signal. All those pathways interact with each other,^8^ which means that signal chains may be blocked or stimulated by components of other signal chains. These are referred to as crosstalk between signal chains, and their components have been interpreted as nodes in a network,^9^, ^10^ analogous to transistors in the integrated circuits of a computer. Thus, any protein component that receives a signal from at least two sides, for example, by dimerization, phosphorylation, and association with allosteric effectors, may be part of a logic gate, whose nature depends on the specific effects of the activity. The notion that information is processed suggests that signal chain components do, in fact, interact in this way. It should be noted that interactions between many elements occur simultaneously, whereas, in classical computers, any processing unit (core) performs only one operation at any time point.

In the described view, extracellular information is encoded via cell receptors and is propagated in a coded form by intracellular reactions. Those reactions regulate the usage of genetic material and organize cell growth and division. Information is activated, or active information is silenced, according to the cells' history and multiple signals the cell receives. Decoding occurs when new components are synthesized and delivered to the extracellular space, displayed at the cell surface, or integrated into the machinery of the cell. New gene products serve as cues to inform other cells, building blocks for biological structures, or enzymes catalyzing metabolic processes. The cell membrane represents not only an interface but also a barrier for the diffusion of effectors. Each cell synthesizes its own signal chain proteins, whose composition may vary between cells. Therefore, single cells may contain specific architectures of logic gates and process information in a different way than other cells. In other words, the signal chain network of a particular cell constitutes the program according to which incoming information is processed.

Every cell of an organism contains the same genetic information in the form of DNA provided by its parents. The cell uses this information selectively, dependent on the cell type. For example, a liver cell uses a certain information subset, different from that of neuronal, muscle, or bone‐producing cells. Regulation of this memory usage depends on incoming signals, their propagation, and on‐target elements associated with genes. Selected DNA sequences are copied into working molecules, messenger RNA (mRNA), corresponding to RAM of the computer. mRNA is transported into the cytoplasm and translated into a protein. Taking the cell as analogous to a computer, cell type corresponds to the program that is executed, and cell differentiation corresponds to program switching. However, the cellular processing machine does not write new information into long‐term genetic memory. With a few exceptions, information flow is unidirectional.

In cancer research, receptors and elements of signal chains that are misexpressed and/or mutated vis‐à‐vis the ordinary component have been associated with disease.^11^, ^12^ These observations underscore the biological significance of the signal chain network in cell regulation. In tumorigenic cells, the normal process of signaling is altered, resulting in a corrupted regulation of cell growth and differentiation. To be able to grow, tumor cells not only evade cell cycle regulation and escape immunological defense mechanisms but also attract blood vessels for nutrient and oxygen supply. Tumorigenic cells function in a quasi‐tissue context.

## A logic gate in embryonic development

Crosstalk between signal chains may involve signal reception, propagation, stability of components, and the effect on gene regulation.^13^ To strengthen the interpretation of crosstalk as communication by logic gates, a cell differentiation event—the appearance of a new cell type—is outlined. In the mammalian preimplantation embryo, cells develop either into the trophectoderm (TE) line, ending as the components of the placenta, or alternatively, into the inner cell mass (ICM) line, leading to all tissues of the embryo proper.^14^, ^15^ The fertilized egg cell, surrounded by the zona pellucida, first divides three times, yielding eight cells (blastomeres) while still inside the zona pellucida. These cells, discernible as morphological entities, undergo contraction. Cells stick closer to each other through adherens junctions, whereby the visible boundaries fade. During this process, cells establish a polarity: the inner part of each cell body is in contact with other cells, and the outer part facing the zona pellucida has no cell contacts. Cell contacts are mediated by E‐cadherin, a homophilic protein of the cell membrane, which takes part in adherens junctions. Cell regions without attachment to neighboring cells contain microvilli, protrusions of the outer membrane that contain abundant actin filaments.

Further cell divisions may occur along the polarization axis, giving rise to two polarized daughter cells (symmetric division). Alternatively, divisions perpendicular to the polarization axis give rise to one polarized cell and one cell situated in the interior of the cell cluster (asymmetric division). This inner cell maintains contact with other cells around the entire surface. It is nonpolarized and takes the ICM route. The geometry of symmetric cell divisions may be guided by cadherin molecules of adherens junctions, which determine the positioning of the mitotic spindle.^16^ Internalization of cells was proposed to be driven by their relative contractility.^17^ Oblique division planes, giving rise to partially polarized cells, have not been described.

The differences in cell polarization and positioning send signals to gene regulation. Genes that consolidate differences and lead to further development are activated. Among those, CDX2 is induced in TE cells but not in ICM cells. One of the mediators is angiomotin (AMOT), a component of signal chains. It is present both in polarized and nonpolarized cells but distributed differently. If activated by phosphorylation, it activates other proteins. AMOT also has a binding affinity to actin; if sequestered by actin, it is not available for phosphorylation. Polarized cells contain AMOT in a sequestered form in cell surface regions, where actin is concentrated. In nonpolarized cells, AMOT is not sequestered and thus accessible to activation. The ensuing signal flow inactivates the cascade protein yes‐associated protein 1 (YAP). Active YAP is a necessary coactivator of TEA domain transcription factor 4 (TEAD4) to induce trophoblast‐specific expression of *CDX2*. Inactive YAP does not allow CDX2 induction, and differentiation toward a trophoblast cell type is blocked. Thus, polarized cells contain active YAP, necessary for CDX2 expression, and nonpolarized cells do not. CDX2 initiates further regulative events in the TE differentiation route. The elimination of CDX2 RNA terminates development later, at hatching and implantation, associated with the functional aspects of TE cells.^18^

**Figure 1:**
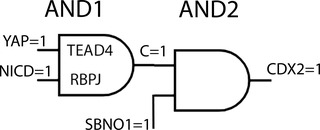
The signal flow through two logic AND gates in the trophectoderm enhancer of CDX2. Prospective trophoblast cells in the >8‐cell embryo contain nuclear targets TEAD4 and RBPJ as part of the AND1 gate. Association of signal chain factors YAP and NICT as the gate input triggers the output C = 1. The ensuing AND2 gate induces transcription of CDX2, if the gate input C = 1 and SBNO1 = 1. Any zero‐input, YAP = 0, NICT = 0, or SBNO1 = 0, will not lead to CDX2 induction.

CDX2 expression also depends on input from NOTCH signaling.^19^, ^20^ The level of NOTCH expression is correlated with the position of the respective cell in the embryo, and it is higher in the outer prospective TE cells than in the inner cells.^21^ Different from AMOT, its distribution is nonpolar. The mechanism that influences NOTCH levels is at present unknown;^21^ profilation by lateral inhibition may be part of it.^20^ NICD, the NOTCH signal (see the section above “The cell as a computation machine”), is released upon ligand binding and targets recombination signal–binding protein for immunoglobulin kappa J region (RBPJ), a factor in the cell nucleus that triggers CDX2 expression. As outlined below, both cell polarity sensing YAP and position sensing NICD contribute to CDX2 induction. The information/technical term for this situation is *the logic AND gate* (Fig. 1). In an AND gate, both input signals must be positive to elicit a positive output signal. Any other value gives a zero output. To determine the cell fate in the [>8‐cell] embryo, YAP may either be active (A = 1) or inactive (A = 0), and NOTCH levels may be elevated (B = 1) or low (B = 0).

AND1 statement

input polarity = variable YAP

YAP active = 1, inactive = 0

input position = variable NICD

NICD high = 1, low = 0

AND1 gate

if YAP = 1, NICD = 1 C = 1: CDX2 expression possible

if YAP = 0, NICD = 1 C = 0: CDX2 not expressed

if YAP = 1, NICD = 0 C = 0: CDX2 not expressed

if YAP = 0, NICD = 0 C = 0: CDX2 not expressed

The physical manifestation of the AND gate is the TE enhancer of *CDX2*, a regulatory DNA sequence upstream of the transcription start site, which includes bound TEAD4 and RBPJ proteins, the receptors for YAP and NICD (Fig. 1).

**Figure 2:**
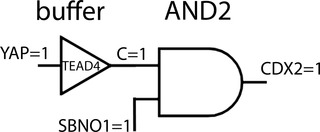
The signal flow through a genetically modified trophectoderm enhancer of CDX2. As in Figure 1, but RBPJ, the target for NICD, is experimentally removed, so that AND1 is abolished and replaced by a buffer gate. The NICD signal is irrelevant. In the mirror experiment, the removal of TEAD4, the NICD signal suffices to induce CDX2.

NOTCH signaling had earlier been refuted as a factor mediating the TE/ICM decision because KO‐mice without RBPJ, the target and transcriptional effector of NOTCH signaling, developed normally to midgestation, far beyond implantation.^22^ NOTCH signaling would, therefore, be dismissible. If the enhancer constitutes the AND gate in the wild‐type situation, with RBPJ as an essential component, this AND gate would be eliminated in RBPJ‐null mutants. If TEAD4 is still active, the AND gate is replaced by a buffer gate, with an output value identical to the input value (Fig. 2). In this way, the YAP signal suffices to trigger CDX2. CDX2 expression in the absence of RBPJ may even be favored if RBPJ, associated with no or other ligands than NICD, acts as a repressor (OFF without NICD and ON if NICD is bound). Such a repressor function of RBPJ has been observed, although in a different context.^23^ Also, for TEAD4, a repressive role in transcription was suggested.^24^ In the meantime, NOTCH signaling as an effector has regained appreciation because NOTCH can rescue CDX2 expression in TEAD4‐deficient TE cells in an artificial reporter construct.^19^ NOTCH/RBPJ rescue does not work at the genome level,^25^ suggesting further so far unknown functions of TEAD4 in a chromatin context. In sum, the test for NOTCH signaling by deleting RBPJ was inconclusive.

**Figure 3:**
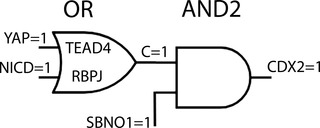
The signal flow through an alternative trophectoderm enhancer structure of CDX2. As in Figure 1, but the AND1 is replaced by an OR gate, describing redundant regulation of CDX2. Either YAP = 1 or NICD = 1 yield C = 1 and induce CDX2 with SBNO1 = 1. The OR gate model is refuted, because it does not account for nonpolarized peripheral cells in the 16‐cell embryo.

The parallel induction of CDX2 by NOTCH and YAP signaling has been understood as redundant regulation, providing robustness to an important biological event.^26^ In logic gate terminology, redundancy corresponds to the OR gate: at least one of the two input signals elicits a positive output (Fig. 3). The expected outcome of elimination of either inducer would be similar to the scenario described above. However, an additional observation suggests that the AND gate is preferable: during the 16‐cell stage, single cells were observed in about 50% of the embryos, which were positioned at the periphery of the embryo but not polarized with respect to AMOT. Most of those cells ended in the ICM.^15^ This finding indicates an important signal from polarization; it is at odds with redundant regulation or an OR‐gate scenario in which peripheral cells are expected to express CDX2 regardless of their polarization. The AND gate model accounts for these cells and formulates an explanation for their behavior: if either the position or polarization signal is missing, CDX2 is not induced, and the cell integrates into the ICM (Fig. 1). A minority of these cells did enter the TE, but those had polarized slowly.^15^ The latter observation indicated a cooperative action of YAP and NICD;^15^, ^20^, ^27^ however, the missing phenotype of the RBPJ‐null mutant (abolished NICD signaling) remained unexplained.

Taken together, if a cell is polarized and positioned at the periphery, it activates CDX2 and develops into a TE cell; any other cell takes the ICM route. Models proposing straight cooperation of YAP and NICD for CDX2 induction do not account for the missing phenotype of the RBPJ‐null mutant because this cooperation requires NOTCH signaling. On the other hand, models proposing YAP or NICD as alternatives for CDX2 induction provide no explanation for peripheral cells with an ICM fate. The gate model accounts for both observations: YAP and NICD cooperate in the wild‐type situation, that is, where both signal targets are present, as the AND gate. In the absence of RBPJ, the AND gate becomes a buffer gate, and cooperation is set aside.

In addition to the described factors, the ubiquitous chromatin component, strawberry NOTCH homolog 1 (SBNO1), was shown as necessary for CDX2 expression.^27^ It directly interacts with YAP/TEAD4 and RBPJ/NICT, and it can bind to both DNA and histones, proteins that organize DNA structure. Embryos without SBNO1 do not develop a blastocoel and die before implantation, suggesting that the absence of SBNO1 also affects functions other than acting as the CDX2 enhancer.^27^ Concerning CDX2, it is here assumed that SBNO1 acts downstream of YAP and NICT because the absence of SBNO1 does not interfere with the localization of these factors.^27^ It is also assumed that SBNO1 is a variable component of the TE enhancer that may be present (active) or absent (inactive). With these premises, a further AND gate (AND2) may be invoked downstream of the one described before. One input is SBNO1, and the other one is the output of the RBPJ/TEAD4 gate (Figs. 1, 2, 3). The tentative role of SBNO1 would be to influence the level of CDX2 transcription, that is, the frequency by which RNA polymerase initiates and/or pauses.

AND2 statement

input = variable SBNO1

SBNO1 present = 1, absent = 0

input = variable C

from AND1; C = 1 or C = 0

AND2 gate

if C = 1, SBNO1 = 1 CDX2 expressed

if C = 1, SBNO1 = 0 CDX2 not expressed

if C = 0, SBNO1 = 1 CDX2 not expressed

if C = 0, SBNO1 = 0 CDX2 not expressed

The model discussed here proposes that logic gates can provide a formal explication of experimental results with two interacting signal chains, in particular the missing phenotype of RBPJ mutants and the ICM fate of surface‐positioned cells. An experimental approach to further distinguish between a redundant CDX2 regulation and the AND1 gate is suggested from Figures 1 and 3: the abolition of either YAP or NICD during embryo contraction would arrest CDX2 expression only if the AND gate model is valid. In the case of redundant regulation, both effectors would have to be suppressed to yield the same effect. Concerning the analysis of the behavior and differentiation of cells in general, logic gates offer an approach that integrates different signaling routes in a formal system. Its focus is on networking, which is the most probable way decisions are made in cells. However, conceiving signaling components like logic gates requires that most involved factors are already known, and that the information flow follows a binary mode.

The logic gates in Figure 1 are only a small part of decision making of the early embryo cell to either become a trophoblast or an ICM cell. There are other factors regulating CDX2 expression and several proteins except for CDX2 that are essential for the TE phenotype. In addition, there are several factors that are specific to ICM cells. Extrapolating from CDX2, many logic gates are suggested to initiate and regulate transcription of these genes, that is, the decision of a cell to take one of the two possible routes.

## Information flow in a tissue

Cell differentiation is initiated by signals that prime cells to alter the use of genetic information. This is achieved both by direct contacts and diffusible factors and their concentration gradients.^28^, ^29^ By consequence, the pattern of proteins is changed and, therefore, cell properties. If the expression of signal chain proteins is affected, future signal interpretation will be changed, too. The appearance of tissue‐specific cell properties is preceded by the appearance of anatomic patterns—anatomical structures are recognizable before tissue‐typical functions are established. Proteins containing homeodomains as DNA‐binding sites, homeoproteins, take part in anatomic patterning; cells in different anatomical locations differ in their homeoprotein composition. For example, in preimplantation embryos, the expression of homeoprotein CDX2 is specific to trophoblast cells, and ICM cells do not express it (see above). Homeoproteins are regulators that modulate the expression of other genes in two ways. First, they can bind to specific DNA sequences, be part of the local chromatin landscape, and thus modulate the accessibility of genes for other factors. Second, if not bound, they may interact as pioneer factors with chromatin and open entry sites.^30^ A selective expression of homeoproteins is part of cell differentiation; it influences gene expression, interactions with other cells, and tissue patterning.^31^ Cells that enter new environments integrate by communication with their new neighbors.^32^

A well‐analyzed formation of an anatomic pattern concerns the early development of flies.^33^, ^34^ Patterning initiates with the action of the morphogenic homeoprotein BICOID and involves a complex array of repressor molecules and a dynamic interplay of many components.^34^ In the first developmental phase, the egg's nucleus multiplies to give a homogenous population of about 1000 nuclei. Cell walls are not formed at this stage, and the nuclei migrate to the periphery of the elongated embryo, which still maintains the shape of an egg. In the anterior part, BICOID is produced from maternal RNA. As a consequence of diffusion and turnover, BICOID forms a concentration gradient with high concentration at the anterior end and low concentration at the posterior end. The anterior end will develop the head, and the posterior pole will develop caudal structures. The middle part of the embryo will later give rise to body segments. BICOID induces 66 target genes, whose expression contributes to the development of head structures and for body segmentation. Activation of different target genes occurs along the body axis in accordance with the local BICOID concentration. A recent model describes how this is achieved:^33^ all DNA in cell nuclei is packaged more or less tightly into chromatin structures as a complex with histones and other proteins, and the mode of packaging is gene specific. It constitutes epigenetic information and is implemented before the BICOID gradient comes into action. In order to allow transcription, DNA packaging has to be partially relieved. Except for its role as a transcription factor, BICOID has the capacity to open chromatin structures so that they become accessible for the transcription apparatus. BICOID's target genes have been grouped according to their sensitivity for BICOID activation. Target genes that need high BICOID concentrations become active at the anterior pole only, and target genes that are easier accessible become active elsewhere, too.^33^ A cascade of gene inductions follows due to cell communications, leading to an anatomical structuring of the fly body. Initiated by the effect of BICOID on chromatin, the homogenous population of cell nuclei becomes differentiated, and different embryo regions transcribe different genes. Thus, the primarily analogous BICOID gradient results in yes/no responses in embryonal domains, that is, the gradient is interpreted in a binary fashion. This makes sense because the BICOID‐sensing system is part of the intracellular processing described above, where signals are propagated in the binary mode. It might be possible to formulate gene activation by BICOID as a sequence of logic gates; however, the elements that make chromatin structures gene‐specific, as well as the mechanisms of packaging and unpackaging, are still not clear.

Anatomic patterns are the basis for later development, which may be illustrated by the formation and maintenance of bone in mammals. The bone matrix consists of collagen and minerals secreted by osteoblasts, bone‐forming cells. During development, it is not sufficient that osteoblasts perform typical synthetic actions; this alone would give rise to a nonordered bone callus. In order to synthesize robust macro‐ and microstructures of the skeleton, the activity of osteoblasts is dependent on their anatomical sites,^35^ and the anatomy of the bone is related to the local expression pattern of homeoproteins.^36^, ^37^ Bone growth is directed by general factors like hormone and growth factor stimulations and local factors that specify anatomical fields, such as homeoproteins. The formation of anatomical fields by homeoprotein patterns, followed by building the skeleton, is understood as intercellular networking with skeletal cells as nodes. The network has an analogy in information technology, namely grid computing. In grids, computers are nodes in a nonhierarchic collaborative network that may form a virtual supercomputer. Specific software—middleware—is used to run the grid.^d^ However, the location of the individual computers in a grid is negligible for their activities, whereas the location of cells in the bone is of pivotal importance for bone synthesis. The cell network is run by cell communication involving the regulation of homeoprotein expression.

Another functional task of the bone cell network is the homeostasis of adult bone, by which the activities of building (osteoblasts) and degrading (osteoclasts) cells are coordinated. Bone tissue adapts permanently to mechanical stress by local synthesis and removal of the bone matrix. Mechanical stress is sensed via small deformations in the bone matrix by osteocytes, cells that are distributed throughout the bone tissue and connected by long protrusions.^38^, ^39^ Osteocytes code mechanical signals into instructions for local osteoblasts and osteoclasts and into the recruitment of precursor cells. The input signals are mechanical stress and anatomical position, and the output reaction is apposition and/or resorption of the bone matrix, which reinforces or weakens the bone locally. Osteocytes respond to mechanical stress by Ca^++^ spikes,^40^ and the communication between osteocytes proceeds via glutamate, a neurotransmitter. This suggests a signaling code of membrane potential pulses and synapse‐like structures.^41^ In terms of computer grids, bone cells correspond to computers, Ca^++^ spike generation corresponds to middleware, and bone apposition and removal to the task to be performed. The anatomical position may modulate cellular responses mediated by the homeoprotein pattern.

Intercellular networks also coordinate tissue repair. Tissue damage emits signals that trigger repair reactions. For example, blood clots initiate a sequence of events, where the differentiation state of cells is altered, and new cells are in many cases recruited to the lesion.^42^ These cells differentiate and build the new ECM, with the aim that the original tissue is reestablished.^43^, ^44^

## Neural tissues

The functional task of neural tissue is data transfer, processing, and storage. The neuron as the dominant cell type is the node in the tissue network. Neurons have an anatomical identity because brain functions can be mapped to anatomical locations. Information enters neural tissue via sensory cells, that is, neurons that may be stimulated by light, heat, taste, or mechanical effects. Sensory cells encode signals into reversible changes of their membrane potential in the form of pulses (spikes) that are fed into the neural network.^45^ Neurons in the ganglia and brain receive such pulses and communicate secondary pulses to neurons or other cells via synapses, particularly cell attachment regions. In the synapse, neurotransmitters (small molecules) secreted from a spiking neuron induce an immediate potential change in the receiver membrane. A single neuron may embody up to 10^4^ synapses. Since neurons send out dendrites and axons, such communication may occur in the axon range; in humans, up to a 1‐m distance. It is much faster than communication by modulation of cell surface determinants or humoral factors. If enough neurons are thus connected, they create an information processing system.^46^ Neural tissue is capable of storing information (memory), which depends on modulations of synapse strengths.^47^, ^48^ Any individual neuron can receive and send spikes, but the pattern of emission differs from that of reception. The input depends on the strength and location of synapses, representing stored information (memory). Output spiking depends on an integration of both excitatory and inhibitory input spikes; the neuron may thus be compared with a leaky electric capacitor.^e^ For some functions, neurons have been compared with logic gates.^46^ However, a spiking decision, in general, requires multiple bits of input information, which suggests that many logic gates must contribute to a cells' decision making. In this case, the neuron corresponds to a computing machine running a program rather than to a single logic gate. As shown for the mouse brain, the population of neurons is highly heterogenous.^49^, ^50^ Neurons are, therefore, likely to run individual programs. The assembly of neurons, that is, the neural tissue, may thus be compared with a supercomputer made of 10^11^ grid computers in the case of the human brain. The neuron's program is materialized in the location and quality of the synapses. It should be noted that the signal code is not an impulse as such, but the frequency of impulses and/or their precise timing.^f^

Neural systems mediate the organism's communication with its surroundings. Simple effects of this informational chain are avoidance responses. Sophisticated reactions, observed among vertebrates and mollusks, are deception of predators, strategies to find prey and mating partners, behavior interpreted as curiosity and collaborative hunting, and human speech. Neural systems have been selected according to the demands that their carrier‐organisms are exposed to. Behavior qualified by us as sophisticated may be observed in animals considered comparatively simple, for example, referential gesture in hunting by fish^51^ or analytical capacities in crows.^52^ Evolution has optimized a degree of flexibility that corresponds to the organisms' demands, including their capability to learn. The neural system manages the information flow between the carrier organism and the outer world.

Neurons develop from nonneuronal precursor cells, and neural tissue executes all information processing related to tissue growth and development like all other tissues. It also feeds back information to nonneural organs, both via synapses that stimulate muscle cells and the humoral system and via the pineal and pituitary glands. In sum, neurons unify two systems of information processing: the developmental network based on biochemical contacts (including diffusible effectors) and the network communicating via membrane potential pulses. The two systems are interconnected. First, the membrane potential network depends on the pattern of synapses,^53^ which is provided by the developmental network. Second, the survival of neurons depends on their electrophysiological activity.^54^ Apoptosis of neurons, executed by the developmental network, is frequent during development.^55^

## Information processing in communication between human beings

The nervous system coordinates organs and regulates behavior of the entire organism, above the level of cells and tissues. Still, there is another outer processing system with individuals as nodes that organizes human societies. It has been argued repeatedly that human communication has characteristics that allow and perform information processing. In fact, network similarities between brains and social structures have been described. Similarities between social neighbor structures and brain cell contacts are used to learn about communication routes in the brain.^56^ Each individual experiences an information input in the form of talking, speech, contracts, laws, patents, and nonverbal communication. In the individual, this information is subjected to previous experience and personal interest that was established beforehand, analogous to a computer program. By communication between individuals, the input information may elicit an output with unforeseen consequences. For example, the agricultural prize regulation in the EU was intended in the 1950s to facilitate life for European food producers. It had, however, severe repercussions on EU policies ever since, and important influences both on European and developing countries, including migration activities. In the view proposed here, “the system” has processed the initial information and created output results that were dependent not only on the input information, that is, the implementation of subsidizing rules, but also on other preexisting interrelations in human communities. We have no good model of the information processing involved, except that we know that information is coded into a language, and decisions are usually graded and not of a yes‐no type. The high degree of sophistication in modern humans has shaped the information processing system of its own right, superior to the individual. Any relevant input will affect the life of not only those who are directly concerned but also of other humans as well as the living world in general.

## Outlook

Electronic devices have been developed from electric switches (on‐off) because of their capability to perform Boolean operations. This system has turned out as most versatile so that almost all information technology today works by means of logic gates and Boolean logic, the basic hardware construction. Signal chains in cells are a product of evolution. They operate by virtue of components that are active or inactive, also suggesting that this communication is binary. The coincidence in binary signaling, necessary for Boolean logic, seems to be the most efficient way to coordinate signals from different provenience into one processing unit. Cells possess only one biochemical organization to store genetic information and to make use of it. DNA, RNA, histone proteins, and other components of the gene expression apparatus are highly conserved in all eukaryotes. On the other hand, there are input signals of various biochemical nature that need to address genetic information. In order to obtain access to the cell's genetic memory, those external signals are coded into a single “genome‐owned” biochemical system. A different physical basis between electronic and cellular information processing notwithstanding, the results from the section “A logic gate in embryo development” suggest that further logic gates will be revealed in biological systems, and that biological interpretations will profit from our skills in electronic programming. The inclusion of artificial neural networks, where graded signals may be handled both as input and output, may extend this approach. The ways by which logic gates are connected in self‐learning programs are often obscure, but methods are being devised by which the data output is mapped to the input, and rules are extracted according to which the network arrives at its results.^57^, ^58^ It seems probable that similar approaches will also help to describe information processing in biological networks.

The access to networks having cells or human individuals as nodes appears to be different. In bone, the cooperation between cells seems to work similarly to middleware. Boolean rules only apply to the most basic intracellular information processing, and cell coordination represents a higher‐level element. In neural tissues, information is transmitted by qualitative aspects of spikes, timing, and frequency. In addition, neurons integrate exciting and inhibiting signals before propagating spikes. This suggests that spike generation does not follow Boolean rules, and alternatively, that our knowledge is not sufficient to discern the basic steps of information processing. Signal transfer in societies works by acoustical or optical means or radiowaves, that is, communications where direct physical contacts between nodes are unnecessary. Also, here, information transfer seems not to be restricted to the binary mode.

Life developed from molecular interactions that amplify and degrade macromolecules. The building plan of living cells, multicellular organisms, and biological functions, such as photosynthesis and metabolism, emerged during evolution. New information was gained during this time by unexpected events (e.g., mutations) provoked by chemical and radiation damage of genetic material.^g^ Precellular assemblies, cells, tissues, organisms, and human societies all have developed information processing. Via their carriers, processing systems are subject to natural selection. Since eukaryotic cells have existed for about 2 billion years, but human societies with sophisticated communications for only 5000 years, information processing in societies is in an early stage of development. Human decision making is notoriously error‐prone if we compare it with the functionality of cells.

Knowledge of logic connections in information technology is far more advanced than in biological research, where components essential for the regulation and construction of tissues still need to be identified and characterized. On the other hand, all biological processors, from the intracellular signal chain network to human societies, handle multiple processing events in parallel, unlike conventional computers. The way cells proceed is unknown; processing seems to be determined by the composition of signal chain elements and their spatial coordination, both influencing the arrangement of logic gates. It may in the future be possible to simulate the cell's processing system in a hardware description language, by which logic gate assemblies may be altered.

## Competing interests

The author declares no competing interests.

## Footnotes

a
https://en.wikipedia.org/wiki/World_Brain

b
https://en.wikipedia.org/wiki/Global_brain

c
http://neuralnetworksanddeeplearning.com/

d
https://en.wikipedia.org/wiki/Grid_computing

e
https://neuronaldynamics.epfl.ch/online/index.html

f
https://en.wikipedia.org/wiki/Neural_coding

g
https://en.wikipedia.org/wiki/Entropy_(information_theory)

## References

[1] Miyamoto, T. , S. Razavi , R. DeRose & T. Inoue . 2013. Synthesizing biomolecule‐based Boolean logic gates. ACS Synth. Biol. 2: 72–82. 23526588 10.1021/sb3001112 PMC3603578

[2] Farnsworth, K. , J. Nelson & C. Gershenson . 2013. Living is information processing: from molecules to global systems. Acta Biotheor. 61: 203–222. 23456459 10.1007/s10441-013-9179-3

[3] Tkacik, G. & W. Bialek . 2016. Information processing in living systems. Annu. Rev. Condens. Matter Phys. 7: 89–117.

[4] Heylighen, F. & J. Bollen . 1996. The world‐wide web as a super‐brain: from metaphor to model. In: Cybernetics and Systems' 96. R. Trappl, Ed.: 917–922. Austrian Society For Cybernetics.

[5] Marks, F. , U. Klingmüller & K. Müller‐Decker . 2017. Cellular Processing: An Introduction to the Molecular Mechanisms of Signal Transduction. 2nd ed. New York and Abington: Taylor & Francis Group.

[6] Csizmok, V. , A.V. Follis , R.W. Kriwacki & J.D. Forman‐Kay . 2016. Dynamic protein interaction networks and new structural paradigms in signaling. Chem. Rev. 116: 6424–6462. 26922996 10.1021/acs.chemrev.5b00548 PMC5342629

[7] Culyba, M.J. 2019. Ordering up gene expression by slowing down transcription factor binding kinetics. Curr. Genet. 65: 401–406. 30353359 10.1007/s00294-018-0896-7 PMC6421095

[8] Fey, D. , D. Matallanas , J. Rauch , *et al*. 2016. The complexities and versality of the RAS‐to‐ERK signalling system in normal and cancer cells. Semin. Cell Dev. Biol. 58: 96–107. 27350026 10.1016/j.semcdb.2016.06.011

[9] Guo, X.X. , S. An , Y. Yang , *et al*. 2018. Emerging role of the Jun N‐terminal kinase interactome in human health. Cell Biol. Int. 42: 756–768. 29418027 10.1002/cbin.10948

[10] Thomaseth, C. , D. Fey , T. Santra , *et al*. 2018. Impact of measurement noise, experimental design, and estimation methods on molecular response analysis based network reconstruction. Sci. Rep. 8: 16217–16231. 30385767 10.1038/s41598-018-34353-3 PMC6212399

[11] Rädler, P.D. , B.L. Wehde & K.U. Wagner . 2017. Crosstalk between STAT5 activation and PI3K/AKT functions in normal and transformed mammary epithelial cells. Mol. Cell. Endocrinol. 451: 31–39. 28495456 10.1016/j.mce.2017.04.025 PMC5515553

[12] Meng, Z. , T. Moroishi & K.L. Guan . 2016. Mechanisms of Hippo pathway regulation. Genes Dev. 30: 1–17. 26728553 10.1101/gad.274027.115 PMC4701972

[13] Luo, K. 2017. Signaling cross talk between TGF‐beta/Smad and other signaling pathways. Cold Spring Harb. Perspect. Biol. 9: a022137. 27836834 10.1101/cshperspect.a022137 PMC5204325

[14] Menchero, S. , T. Rayon , M.J. Andreu & M. Manzanares . 2017. Signaling pathways in mammalian preimplantation development: linking cellular phenotypes to lineage decisions. Dev. Dyn. 246: 245–261. 27859869 10.1002/dvdy.24471

[15] Sasaki, H. 2017. Roles and regulation of HIPPO signaling during preimplantation mouse development. Dev. Growth Differ. 59: 12–20. 28035666 10.1111/dgd.12335

[16] Gloerich, M. , J.M. Bianchini , K.A. Siemers , *et al*. 2017. Cell division orientation is coupled to cell–cell adhesion by the E‐cadherin/LGN complex. Nat. Commun. 8: 13996–14007. 28045117 10.1038/ncomms13996 PMC5216124

[17] Maître, J.L. , H. Turlier , R. Illukkumbura , *et al*. 2016. Asymmetric division of contractile domains couples cell positioning and fate specification. Nature 536: 344–348. 27487217 10.1038/nature18958 PMC4998956

[18] Wu, G. , L. Gentile , T. Fuchikami , J. Sutter , *et al*. 2010. Initiation of trophectoderm lineage specification in mouse embryos is independent of Cdx2. Development 137: 4159–4169. 21098565 10.1242/dev.056630 PMC2990207

[19] Rayon, T. , S. Menchero , A. Nieto , *et al*. 2014. Notch and Hippo converge on Cdx2 to specify the trophectoderm lineage in the mouse blastocyst. Dev. Cell 30: 410–422. 25127056 10.1016/j.devcel.2014.06.019 PMC4146744

[20] Totaro, A. , M. Caastellan , D. Di Biagio & S. Piccolo . 2018. Crosstalk between YAP/TAZ and Notch signaling. Trends Cell. Biol. 28: 560–573. 29665979 10.1016/j.tcb.2018.03.001 PMC6992418

[21] Menchero, S. , I. Rollan , A. Lopez‐Izquierdo , *et al*. 2019. Transitions in cell potency during early mouse development are driven by Notch. eLife 8. e42930. 30958266 10.7554/eLife.42930 PMC6486152

[22] Souilhol, C. , S. Cormier , K. Tanigaki , *et al*. 2006. RBP‐J kappa‐dependent notch signaling is dispensable for mouse early embryonic development. Mol. Cell. Biol. 26: 4769–4774. 16782866 10.1128/MCB.00319-06 PMC1489163

[23] Yuan, Z. , B.D. VanderWielen , B.D. Giaimo , *et al*. 2019. Structural and functional studies of the RBPJ–SHARP complex reveal a conserved corepressor binding site. Cell. Rep. 26: 845–854. 30673607 10.1016/j.celrep.2018.12.097 PMC6352735

[24] Frum, T. , J.L. Watts & A. Ralston . 2019. TEAD4, YAP1 and WWTR1 prevent the premature onset of pluripotency prior to the 16‐cell stage. Development 146. dev179861. 31444221 10.1242/dev.179861 PMC6765126

[25] Yagi, R. , M.J. Kohn , I. Karavanova , *et al*. 2007. Transcription factor TEAD4 specifies the trophectoderm lineage at the beginning of mammalian development. Development 134: 3827–3836. 17913785 10.1242/dev.010223

[26] Frum, T. & A. Ralston . 2015. Cell signaling and transcription factors regulating cell fate during formation of the mouse blastocyct. Trends Genet. 31: 402–410. 25999217 10.1016/j.tig.2015.04.002 PMC4490046

[27] Watanabe, Y. , K.Y. Miyasaka , A. Kubo , *et al*. 2017. Notch and Hippo signaling converge on Strawberry Notch 1 (Sbno1) to synergistically activate Cdx2 during specification of the trophectoderm. Sci. Rep. 7: 46135–46152. 28401892 10.1038/srep46135 PMC5389439

[28] Pinto, P.B. , J.M. Espinosa‐Vázquez , M.L. Rivas & J.C. Hombría . 2015. JAK/STAT and Hox dynamic interactions in an organogenetic gene cascade. PLoS Genet. 11. e1005412. 26230388 10.1371/journal.pgen.1005412 PMC4521708

[29] Briscoe, J. & S. Small . 2015. Morphogen rules: design principles of gradient‐mediated embryo patterning. Development 142: 3996–4009. 26628090 10.1242/dev.129452 PMC4712844

[30] Grebbin, B.M. & D. Schulte . 2017. PBX1 as pioneer factor: a case still open. Front. Cell Dev. Biol. 5: 9. 28261581 10.3389/fcell.2017.00009 PMC5306212

[31] Merabel, S. & R. Mann . 2016. To be specific or not: the critical relationship between Hox and TALE proteins. Trends Genet. 32: 334–347. 27066866 10.1016/j.tig.2016.03.004 PMC4875764

[32] Nieto, M.A. , R.Y.‐J. Huang , R.A. Jackson & J.P. Thiery . 2016. Emt: 2016. Cell 166: 21–45. 27368099 10.1016/j.cell.2016.06.028

[33] Hannon, C.E. , S.A. Blythe & E.F. Wieschaus . 2017. Concentration dependent chromatin states induced by the bicoid morphogen gradient. eLife 6. e28275. 28891464 10.7554/eLife.28275 PMC5624782

[34] Surkova, S. , E. Golubkova , L. Mamon & M. Samsonova . 2018. Dynamic maternal gradients and morphogenic networks in *Drosophila* early embryo. BioSystems 173: 207–213. 30315821 10.1016/j.biosystems.2018.10.009

[35] Wurtz, T. & A. Berdal . 2003. Osteoblast precursors at different anatomic sites. Crit. Rev. Eucaryot. Gene Expr. 13: 147–161. 10.1615/critreveukaryotgeneexpr.v13.i24.80 14696963

[36] Wellik, D.M. & M.R. Capecci . 2003. Hox10 and Hox11 genes are required to globally pattern the mammalian skeleton. Science 301: 363–367. 12869760 10.1126/science.1085672

[37] Ishii, M. , A.E. Merrill , Y.‐S. Chan , *et al*. 2003. Msx2 and Twist cooperatively control the development of the neural crest‐derived skeletogenic mesenchymeof the mural skull vault. Development 130: 6131–6142. 14597577 10.1242/dev.00793

[38] Burger, E.H. & J. Klein‐Nulen . 1999. Responses of bone cells to biomechanical forces *in vitro* . Adv. Dent. Res. 13: 93–98. 11276754 10.1177/08959374990130012201

[39] Chen, J.H. , C. Liu , L. You & C.A. Simmons . 2010. Boning up on Wolff's Law: mechanical regulation of the cells that make and maintain bone. J. Biomech. 43: 108–118. 19818443 10.1016/j.jbiomech.2009.09.016

[40] Jing, D. , A.D. Baik , X.L. Lu , *et al*. 2014. *In situ* intracellular calcium oscillations in osteocytes in intact mouse long bones under dynamic mechanical loading. FASEB J. 28: 1582–1592. 24347610 10.1096/fj.13-237578 PMC3963014

[41] Mason, D.J. 2004. The role of glutamate transporters in bone cell signalling. J. Musculoskelet. Neuronal Interact. 4: 128–131. 15615110

[42] Barker, T.H. & A.J. Engler . 2017. The provisional matrix: setting the stage for tissue repair outcomes. Matrix Biol. 60–61: 1–4. 10.1016/j.matbio.2017.04.003 PMC5831186 28527902

[43] Reinke, J.M. & H. Sorg . 2012. Wound repair and regeneration. Eur. Surg. Res. 49: 35–43. 22797712 10.1159/000339613

[44] Etulain, J. 2018. Platelets in wound healing and regenerative medicine. Platelets 29: 556–568. 29442539 10.1080/09537104.2018.1430357

[45] Zhou, S. & Y. Yu . 2018. Synaptic E/I balance underlies efficient neural coding. Front. Neurosci. 12: 46–57. 29456491 10.3389/fnins.2018.00046 PMC5801300

[46] Koch, C. & I. Segev . 2000. The role of single neurons in information processing. Nat. Neurosci. 3(Suppl.): 1171–1177. 11127834 10.1038/81444

[47] Pinho, J. , C. Marcut & R. Fonseca . 2020. Actin remodeling, the synaptic tag and the maintenance of synaptic plasticity. IUMB Life 72: 577–589. 10.1002/iub.2261 32078241

[48] Bennett, S.H. , A.J. Kirby & G.T. Finnerty . 2018. Rewiring the connectome: evidence and effects. Neurosci. Biobehav. Rev. 88: 51–62. 29540321 10.1016/j.neubiorev.2018.03.001 PMC5903872

[49] Pestana, F. , G. Edwards‐Faret , T. Belgard , *et al*. 2020. No longer underappreciated: the emerging concept of astrocyte heterogeneiety in neuroscience. Brain Sci. 10: 168–189. 10.3390/brainsci10030168 PMC7139801 32183137

[50] Zeisel, A. , H. Hochgerner , P. Lönnerberg , *et al*. 2018. Molecular architecture of the mouse nervous system. Cell 74: 999–1014. 10.1016/j.cell.2018.06.021 PMC6086934 30096314

[51] Vail, A.L. , A. Manica & R. Bshary . 2013. Referential gestures in fish collaborative hunting. Nat. Commun. 4: 1765–1772. 23612306 10.1038/ncomms2781

[52] Balakhonov, D. & J. Rose . 2017. Crows rival monkeys in cognitive capacity. Sci. Rep. 7: 8809–8817. 28821812 10.1038/s41598-017-09400-0 PMC5562807

[53] Wilton, D.K. , L. Dissing‐Olessen & B. Stevens . 2019. Neuron–glia signaling in synapse elimination. Ann. Rev. Neurosci. 42: 107–127. 31283900 10.1146/annurev-neuro-070918-050306

[54] Hutchins, J.B. & S.W. Barger . 1998. Why neurons die: cell death in the nervous system. Anat. Rec. 253: 79–90. 9700393 10.1002/(SICI)1097-0185(199806)253:3<79::AID-AR4>3.0.CO;2-9

[55] Fricker, M. , A.M. Tolkovsky , V. Borutaite , *et al*. 2018. Neuronal cell death. Physiol. Rev. 98: 813–880. 29488822 10.1152/physrev.00011.2017 PMC5966715

[56] Falk, E.B. & D.S. Bassett . 2017. Brain and social networks: fundamental building blocks of human experience. Trends Cogn. Sci. 21: 674–690. 28735708 10.1016/j.tics.2017.06.009 PMC8590886

[57] Mereani, F. & J.M. Howe . 2019. Exact and approximate rule extraction from neural networks with Boolean features. In Proceedings of the 11th Joint Conference on Comput*ational* Intelligence, 424–433.

[58] Haileselassie, T. 2016. Rule extraction algorithm for deep neural networks: a review. IJCSIS 14: 376–381.

